# Insomnia contributes to paranoid thoughts through mechanisms involving anxiety and non-constructive rumination

**DOI:** 10.1038/s41598-026-41689-8

**Published:** 2026-02-27

**Authors:** Julie Faccini, Damien Vistoli, Fabio Cannas-Aghedu, Jonathan Del-Monte

**Affiliations:** 1 Clinical Research Unit, Délégation à la Recherche Clinique et à l’Innovation du Groupement Hospitalier de Territoire du Var, Centre Hospitalier Intercommunal Toulon La Seyne-sur-Mer, 54 Rue Henri Sainte-Claire Deville, Toulon, France; 2 Laboratoire ENACT, Université de Nîmes, Nîmes, France; 3 Laboratoire d’Anthropologie et de Psychologie Clinique, Cognitive et Sociale (LAPCOS, UPR 7278), Université Côte d’Azur, Nice, France

**Keywords:** Diseases, Psychology, Psychology

## Abstract

Insomnia is a significant public health concern associated with cognitive difficulties and mental health problems. Growing evidence suggests that anxiety and rumination may explain how insomnia contributes to paranoid thoughts. Yet, the specific roles of non-constructive rumination and the distinction between daytime and nighttime insomnia symptoms remain unclear. This study investigated the relationships among insomnia symptoms, anxiety, rumination styles, and paranoid thoughts in adults with and without insomnia. A total of 486 French-speaking participants completed self-report measures assessing insomnia, anxiety, rumination, and paranoid ideation. Network analyses identified the most central symptoms and their associations, and mediation analyses examined whether anxiety and non-constructive rumination explained the link between insomnia and paranoia. Among participants with insomnia, anxiety, non-constructive rumination, and daytime symptoms were strongly connected to paranoid thoughts, and both anxiety and non-constructive rumination partially mediated their relationship, with anxiety showing the strongest influence. However, no causal relationships can be concluded. In contrast, these associations were absent in individuals without insomnia. These findings suggest that daytime symptoms of insomnia may foster paranoid thinking through heightened anxiety and repetitive negative thinking. Understanding these mechanisms highlights the importance of addressing emotional regulation and maladaptive rumination in the treatment of insomnia and related paranoid ideation.

**Supplementary Information:**

The online version contains supplementary material available at 10.1038/s41598-026-41689-8.

## Introduction

Insomnia, characterized by dissatisfaction with sleep quality or duration, is a major public health concern commonly reported in primary care^1^. It is more prevalent among women and among individuals with medical or psychiatric conditions^2^. According to DSM-5, chronic insomnia is defined by difficulties initiating or maintaining sleep and/or early awakenings at least three times per week for three months or more^3^. Insomnia is also characterized by daytime symptoms such as fatigue, cognitive impairments, and mood disturbances, which help clarify the pathophysiology of insomnia as well as the associated risks.^4^. Sleep plays a crucial role in emotion regulation, which may explain its strong link with anxiety: 70–80% of anxious individuals, experiencing overwhelming negative emotions, report insomnia^5^. Daytime symptoms show stronger associations with anxiety than nighttime ones^6,7^. Harrington & Cairney proposed that insomnia-related sleep deprivation disrupts memory suppression, fostering intrusive thoughts and emotional dysregulation. This is complementary to Harvey’s model^8^, in which dysfunctional cognitions and anxious arousal perpetuate insomnia in a vicious cycle. Among these cognitions, paranoid thoughts are especially detrimental.

Paranoia, an irrational fear of harm by others^9^, occurs on a continuum, from psychotic disorders to prevalence rates in 15–20% of the general population^10^^11^. Freeman’s model^12^^13^ suggests paranoia arises from interactions between cognitive and emotional pathways, with sleep disturbances amplifying negative affect, dysregulation, and unusual experiences. Prior studies confirm associations between insomnia and paranoia^14^^15^, mediated by negative emotions (such as anxiety) and rumination^16^^17^.

The literature increasingly distinguishes between two types of rumination: constructive rumination, characterized by a concrete, problem-solving style, and non-constructive rumination, characterized by an abstract, repetitive, and non-resolving focus on distress. Constructive rumination can facilitate emotional processing and lead to actionable insights or adaptive coping strategies. In contrast, non-constructive rumination often involves circular thinking about causes and consequences without resolution, and is particularly harmful to mental health^18^^19^. This type of cognition has been highlighted as playing a mediating role between insomnia and the emergence of other dysfunctional thoughts, like maladaptive beliefs about sleep^20^.

Yet, the combined role of insomnia, paranoia, anxiety, and non-constructive rumination on paranoid remains unexplored.

Network models provide a relevant framework for studying the relationships between symptoms and can offer useful clinical insights^6^. In this context, the objectives of the study were to identify the overall network structure of both daytime and nighttime insomnia symptoms, anxiety, types of rumination (constructive and non-constructive), and paranoid thoughts in a population with insomnia compared to a population without insomnia. Another objective is to identify mediators of the relationship between insomnia and paranoid thoughts.

Based on existing literature^14^^16^^17^^20^, we hypothesised that: (i) the insomnia-group would exhibit more associations between insomnia symptoms, anxiety, non-constructive rumination, and paranoid thoughts than non-insomnia group ; (ii) non-constructive rumination would mediate the relationship between total insomnia (daytime and nighttime symptoms) and paranoid thoughts in the insomnia group; and (iii) anxiety would act as a mediator of the total insomnia (daytime and nighttime symptoms)–paranoid thoughts relationship in both the insomnia and non-insomnia groups.

## Method

### Participant recruitment and study protocol

The questionnaire was developed using Qualtrics and disseminated via social media platforms (Facebook, WhatsApp, LinkedIn) between September 10 and December 15, 2024. Participants were informed of the study’s purpose, procedures, data analysis, the voluntary nature of their participation, confidentiality, objectives, methods, institutional affiliations, expected benefits, potential risks of the research, and possible inconveniences.

The authors assert that all procedures contributing to this work comply with the ethical standards of the relevant national and institutional committees on human experimentation and with the Helsinki Declaration of 1975, as revised in 2013. All procedures involving human subjects/patients were approved by the Ethics Committee of Aix-Marseille University (approval number 2020012307).

The entire protocol was conducted online. Participants signed an informed consent form, completed the hetero-questionnaires. The total duration of the research protocol was approximately 20 min.

The inclusion criteria were age ≥ 18 years and fluency in French (reading, speaking, and comprehension). Participants who did not complete all questionnaires or provided incongruent responses (e.g., answering “yes” to a question about age) were excluded from the analyses.

A total of 617 valid responses were retained after excluding missing data and invalid responses (*n* = 203). The sample was divided into groups: an insomnia group of 226 participants meeting the criteria for insomnia according to the Sleep Condition Indicator which is a validated scale in a French population for diagnosing insomnia^21^(i.e., all participants scoring equal to or below 16), and a group of 391 participants composed of individuals not suffering from insomnia according to the Sleep Condition Indicator (i.e., all participants scoring above 16). To balance the groups, we performed a random selection of 60% of the participants who did not suffer from insomnia (*n* = 260) using JASP, following the methodology of Andrad et al.^22^. We verified that the groups were well matched in terms of age and sex.

### Measures

The Sleep Condition Indicator (SCI)^21^ is an 8-item measure used to evaluate insomnia symptoms. Each item is rated on a 4-point Likert scale (ranging from 0 to 4), with lower total scores reflecting more severe insomnia. A total score of 16 or below is considered indicative of probable insomnia. Items 1, 2, 3, 4, and 8 focus on nighttime symptoms, while items 5, 6, and 7 address daytime impairments. The French version of the SCI has shown strong internal consistency, with a Cronbach’s alpha of 0.87 reported in the scale’s validation study^22^. In the current sample, internal consistency was nearly equivalent, with a Cronbach’s alpha of 0.84.

The Hospital Anxiety and Depression Scale (HADS)^23^ is used to evaluate the severity of anxiety and depressive symptoms. The French adaptation of this scale includes 14 items, each rated on a scale from 0 to 3. Seven items measure anxiety, while the other seven assess depression. The scale’s authors have established threshold values: scores from 0 to 7 suggest no presence of anxiety or depression; scores from 8 to 10 reflect possible symptoms that warrant further evaluation; and scores of 11 or above indicate a high likelihood of clinically significant symptoms. As a result, subscale scores range from 0 to 21. In the validation study^23^, the internal consistency (Cronbach’s alpha) was reported as 0.78 for the depression subscale and 0.81 for the anxiety subscale. In the present study, Cronbach’s alpha was 0.75 for the depression items and 0.78 for the anxiety items.

The Mini Cambridge-Exeter Repetitive Thought Scale (Mini-CERTS)^24^ is a brief 16-item self-report questionnaire designed to assess both constructive and non-constructive forms of rumination. Responses are rated on a 4-point scale ranging from 1 (almost never) to 4 (almost always). Participants were asked to evaluate how they typically think when faced with challenging situations. The Mini-CERTS has demonstrated acceptable internal consistency, with a Cronbach’s alpha of 0.71. In the present study, Cronbach’s alpha was 0.67.

Paranoid thoughts were assessed using the Green Paranoid Thoughts Scale (GPTS)^25^, a self-report instrument designed to evaluate paranoid thought patterns. The scale comprises 32 items rated on a 5-point Likert scale, ranging from 1 (Not at all) to 5 (Totally). It includes two distinct 16-item subscales: the first measures ideas of social reference associated with paranoia, and the second captures persecutory ideation. Each subscale yields scores between 16 and 80, with higher values indicating greater levels of paranoid ideation and stronger tendencies toward paranoid thinking. In the present study, only the persecution subscale was used. Cronbach’s alpha calculated for the study sample was 0.92.

### Statistical analysis

Data analysis was carried out using JASP (version 0.12.2.0)^26^ and Jamovi (version 2.4.11)^27^. Based on the Shapiro-Wilk test results, the variables did not follow a normal distribution (*p* < 0.05). Correlation analyses were conducted separately for each group to examine the relationships between different insomnia symptoms, anxiety, constructive and non-constructive rumination, and paranoid thoughts.

A network analysis was performed for the insomnia group and non-insomnia group, including the following variables: anxiety, insomnia symptoms (daytime and nighttime), constructive and non-constructive rumination, and paranoid thoughts. In this network, each variable is represented as a “node,” and connections between them, known as “edges,” typically reflect partial correlations. This analytic approach does not support causal interpretations.

To estimate the network structure, we used the graphical Least Absolute Shrinkage and Selection Operator (gLASSO)^28^, a method that applies a regularization penalty to reduce the number of estimated parameters. This approach accounts for both the sample size and model complexity, thereby helping to mitigate the risk of Type I errors due to multiple comparisons. The resulting edges in the network represent regularized partial correlations: the thicker the edge, the stronger the correlation. Edges are color-coded—blue for positive associations and red for negative ones.

The optimal network model was selected using the Extended Bayesian Information Criterion (EBIC)^29^. Three centrality measures were used to assess the role of each variable within the network: strength, betweenness, and closeness. Node strength refers to the sum of the absolute values of all edge weights connected to a given node, indicating its overall influence within the network. A node with high strength is considered to have strong direct interactions with other variables. Betweenness centrality reflects how often a node lies on the shortest path between two other nodes, capturing its function as a connector or intermediary. Closeness centrality measures the average length of the shortest paths from a node to all others, with higher closeness indicating a variable that can rapidly influence the entire network. Expected Influence is a centrality measure to assess the overall impact a node (i.e., a variable) has on the rest of the network. Unlike traditional centrality metrics that only consider the absolute strength of connections, expected influence takes into account both the strength and the direction of the connections. A high positive expected influence indicates a rapid spread of a node’s influence within the network. A high negative expected influence indicates a strong but inhibitory effect on other variables. A value close to zero implies that the positive and negative influences cancel each other out, or the node has minimal impact.

Simple mediation analyses were conducted using Jasp for each group separately. These analyses examined the potential mediating role of anxiety and non-constructive rumination, individually, in the relationship between insomnia and paranoid thoughts. Subsequently, multiple mediation analyses were performed with the GLM Mediation Model module of Jamovi, simultaneously including both anxiety and non-constructive rumination as potential mediators of the association between insomnia and paranoid thinking, for both groups. The proposed direct (c) and indirect effects (ab = a * b) were assessed by estimating bias-corrected standardized regression coefficients (betas) through bootstrap analysis (1000 bootstrap samples), as recommended by Biesanz et al.^30^. A 95% bias-corrected confidence interval (CI) around the indirect effect point estimate that does not pass through zero indicates statistical significance.

## Results

### Descriptive statistics

Participants were matched for age, gender, education level, and marital status, except for the ‘Other’ subcategory within marital status (Table 1).

**Table 1:** Socio-demographic and clinical data and their between-group comparaisons.

	Insomnia Group (*N* = 226)	Non-Insomnia Group (*N* = 260) (M ± SD or %)	χ²/t (df)	*p*
Age	31.08 ± 12.54	32.83 ± 13.76	-1.76	0.079
Gender (% Female)	73.5%	66.9%	2.92	0.818
Marital Status *Single* *Cohabitation/Married* *Other*	53.1% 41.6% 2.3%	48.1% 43.5% 8.4%	1.223 0.179 8.99	0.269 0.673 0.003
Education level (% ≥ Bachelor’s degree)	95.13%	97.31%	1.598	0.206
Anxiety (out of XX)	10.52 ± 3.50	7.60 ± 3.16	9.66(484)	0.176
Depression	6.38 ± 3.80	3.23 ± 2.52	9.85(484)	0.000
Insomnia *Total score* *Daytime symptoms* *Nighttime symptoms*	11.44 ± 3.70 4.62 ± 2.45 5.01 ± 2.51	23.84 ± 4.49 8.46 ± 2.46 9.92 ± 1.87	-32.87(484) 17.17(484) -24.62(484)	0.001 0.921 0.000
Ruminations *Constructive* *Non-constructive*	15.18 ± 3.46 24.29 ± 4.71	16.32 ± 4.08 21.46 ± 4.56	-3.30(484) 6.71(484)	0.045 0.171
Paranoid thoughts	13.05 ± 10.52	8.20 ± 8.70	5.56(484)	0.000

The insomnia group scored significantly higher than the non-insomnia group on all clinical variables, except for anxiety, daytime symptoms, and non-constructive ruminations, for which no significant differences were found. Regarding constructive ruminations, the non-insomnia group had significantly higher scores than the insomnia group (Table 1).

Correlation analyses for both groups are provided in the supplementary material.

### Network analysis

**Fig. 1:**
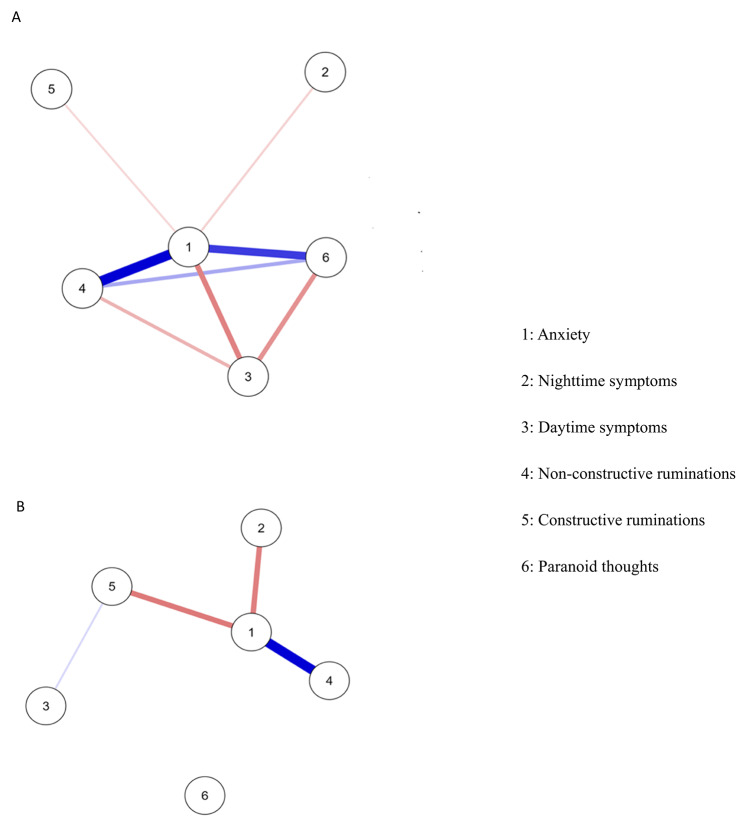
Network analysis for insomnia group (**A**) (*N* = 226) & non-insomnia group (**B**) (*N* = 260). Blue and red edges indicate positive and negative associations, respectively. The thickness of the edges represents the strength of the relationship between two variables.

The network analysis for the insomnia group (Fig. 1a) revealed strong interconnections among all variables, with the exception of nighttime insomnia symptoms and constructive ruminations. Anxiety occupied a central position, bridging non-constructive rumination and paranoid thoughts, and was closely linked to daytime symptoms, while also maintaining weaker direct connections with nighttime symptoms and constructive rumination.

The network analysis further showed that the daytime symptoms node (reverse-scored : indicates a positive relationship) has a strong negative relationship with the nodes for non-constructive ruminations, anxiety, and paranoid thoughts. The anxiety node has strong positive relationships with non-constructive ruminations and paranoid thoughts, and weaker negative relationships with constructive ruminations and nighttime symptoms. The non-constructive ruminations node has a strong, direct, and positive relationship with the paranoid thoughts node, as well as a strong, direct, and negative relationship with the daytime symptoms node.

Finally, Fig. 1a showed that the link between daytime symptoms and constructive ruminations appears to be routed through the anxiety node, which seems to reduce the strength of the connection.

**Fig. 2:**
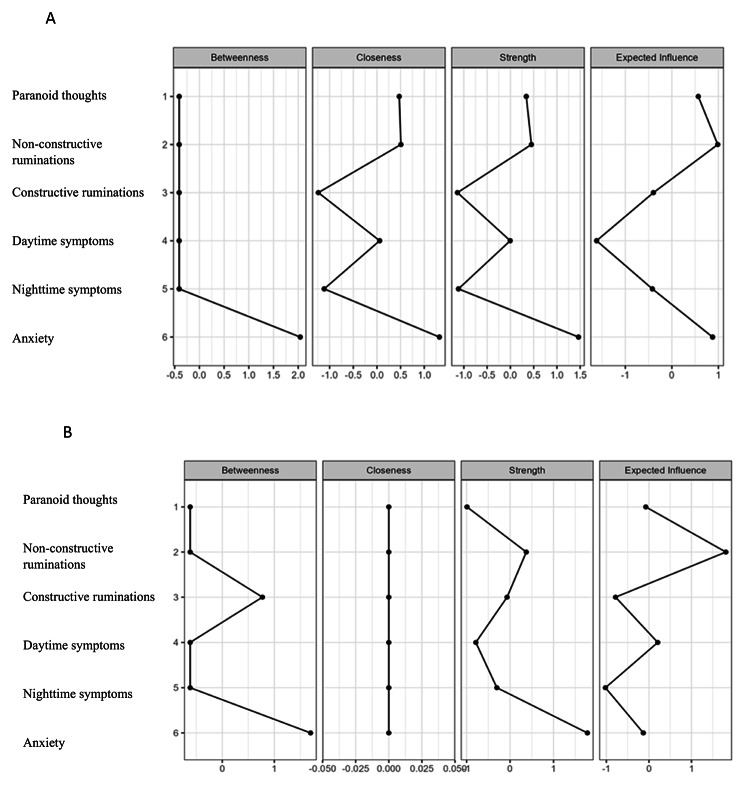
Centrality plot depicting standardized centrality indices (betweenness, closeness, strength, expected influence) for insomnia group (**A**) (*N* = 226) & non-insomnia group (**B**) (*N* = 260).

As shown in Fig. 2a, analyses of centrality indices in insomnia group showed that the highest betweenness centrality was observed for the anxiety node (z = 2.041). For closeness, the strongest nodes were non-constructive ruminations (z = 0.506), paranoid thoughts (z = 0.471), and anxiety (z = 1.319). Regarding strength centrality, non-constructive ruminations (z = 0.986), paranoid thoughts (z = 0.341), and anxiety (z = 1.464) were most prominent. Similarly, expected influence highlighted non-constructive ruminations (z = 0.986), paranoid thoughts (z = 0.568), and anxiety (z = 0.875) as key nodes (Fig. 2).

In the non-insomnia group, network analysis highlighted the central roles of anxiety, non-constructive rumination, nighttime symptoms, and constructive rumination as illustrated in Fig. 1b. Only anxiety and constructive rumination were connected to other nodes. Paranoid thoughts were completely disconnected, and daytime symptoms appeared peripheral. Anxiety was the most connected node, showing strong positive links with non-constructive rumination and strong negative associations with nighttime symptoms (reverse-scored) and constructive rumination. Constructive rumination was weakly and positively linked to daytime symptoms.

Regarding centrality indices for the non-insomnia group (Fig. 2b), the results showed that the nodes with the highest centrality for the betweenness index are anxiety (z = 1.697) and constructive ruminations (z = 0.772). Concerning the closeness index, all variables have the same score (z = 0.000) given that the paranoid thoughts node is isolated (not connected to any other node). Nodes with the highest centrality for the strength index are: « non-constructive ruminations » (z = 0.373), and anxiety (z = 1.778). For the expected influence, the most important nodes were non-constructive ruminations (z = 1.809), and daytime symptoms (z = 0.206).

### Mediation analysis

For the insomnia group, simple mediation analyses were conducted to examine the relationship between total insomnia (predictor) and paranoid thoughts (dependent variable), testing the mediating role of anxiety and non-constructive ruminations.

**Fig. 3:**
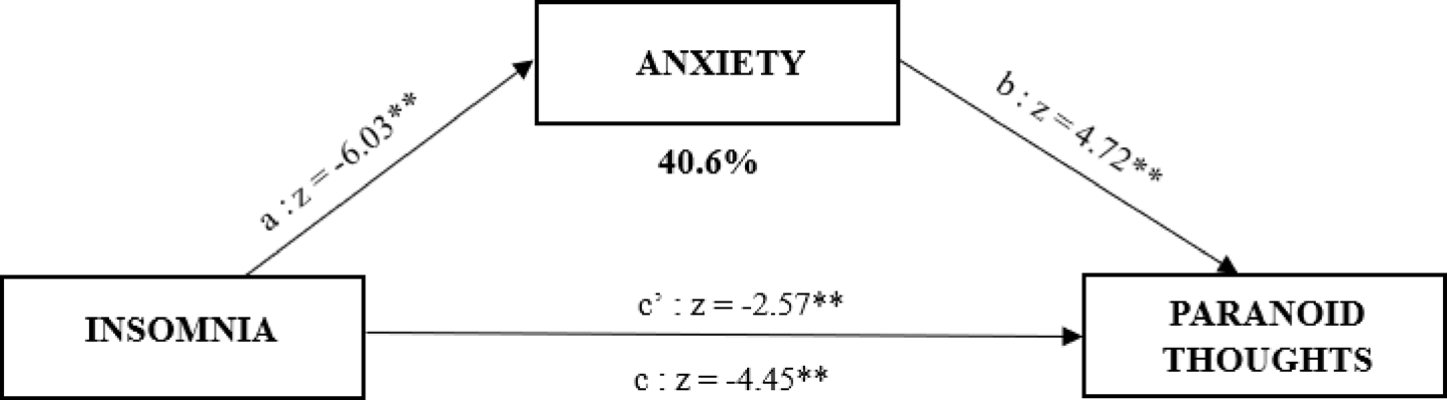
Mediation analysis of anxiety on the relationship between insomnia and paranoid thoughts in the insomnia group (N = 226). C’ = Total Effect; C’ = Direct Effect. * = p.<0.05 ; ** = p.< 0.01.

The first simple mediation analysis showed that both the direct (estimate: -0.479, β = -0.05, *CI*_*0.95*_ = [-0.844 – -0.114], *p* = 0.010) and indirect effect (estimate: -0.328, β = -0.031, *CI*_*0.95*_ = [-0.500 – -0.155], *p* < 0.01) were significant, with anxiety playing an important mediating role in the relationship between insomnia and paranoid thoughts. The mediation effect accounted for 40.6% of the total effect (estimate: -0.807, β = -0.08, *CI*_*0.95*_ = [-1.162– -0.451], *p* < 0.01). (Fig. 3).

**Fig. 4:**
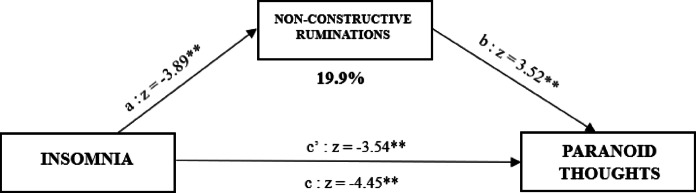
Mediation analysis of non-constructive ruminations on the relationship between insomnia and paranoid thoughts in the insomnia group (N = 226). C’ = Total Effect; C’ = Direct Effect. * = p.<0.05 ; ** = p.< 0.01.

The second simple mediation analysis showed that both the direct (estimate: -0.646, β = -0.06, *CI*_*0.95*_ = [-1.003 – -0.288], *p* < 0.001) and indirect effect (estimate: -0.161, β = -0.01, *CI*_*0.95*_ = [-0.281 – -0.040], *p* = 0.009) were significant, with non-constructive ruminations playing a significant mediating role in the relationship between insomnia and paranoid thoughts. The mediation effect accounted for 19.9% of the total effect (estimate: -0.807, β = -0.07, *CI*_*0.95*_ = [-1.162– -0.451], *p* < 0.01) (Fig. 4).

**Fig. 5:**
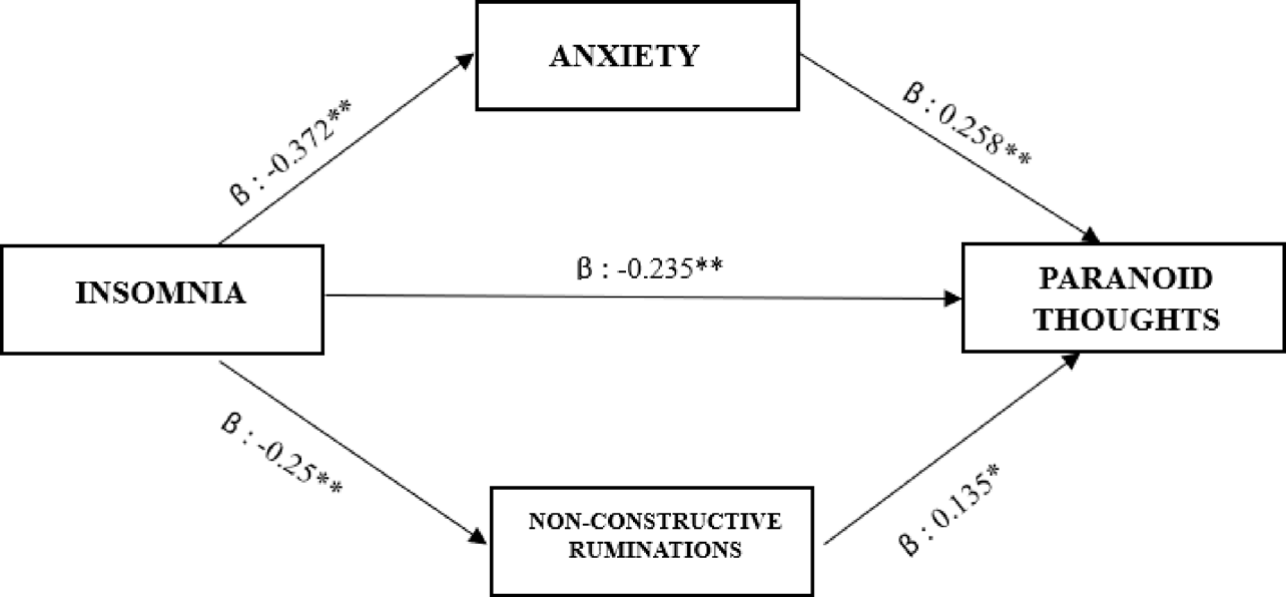
Multiple mediation analysis of anxiety and non-constructive ruminations on the relationship between insomnia and paranoid thoughts in the insomnia group (*N* = 226). * = p.<0.05 ; ** = p.< 0.01.

The multiple mediation analyses simultaneously assessed the mediating roles of anxiety and non-constructive rumination in the relationship between insomnia and paranoid thoughts, showed that the direct (estimate: -0.437, β = -0.153, *CI*_*0.95*_ = [-0.801 – -0.073], *p* = 0.019) and total effect (estimate: -0.806, β = -0.283, *CI*_*0.95*_ [-1.162 – -0.450], *p* < 0.001) was significant. The indirect effect via anxiety was significant (estimate: -0.273, β = -0.096, *CI*_*0.95*_ = [-0.442–0.103], *p*.<0.05). The indirect effect via non-constructive ruminations was not significant (estimate: -0.096, β = -0.033, *CI*_*0.95*_ [-0.201 – -0.009], *p* = 0.073) (Fig. 5). Collinearity among the mediators was assessed, and the results indicated low levels of collinearity, with variance inflation factors (VIFs) of 1.22.

For the non-insomnia group, simple mediation analyses were conducted to examine the relationship between total insomnia (predictor) and paranoid thoughts (dependent variable), testing the mediating role of anxiety and non-constructive ruminations.

The first simple mediation analysis, testing the mediating role of anxiety, showed that the direct (estimate: -0.205, β = -0.02, *CI*_*0.95*_ = [-0.451–0.039], *p* = 0.10), total effect (estimate: -0.024, β = -0.02, *CI*_*0.95*_ [-0.052–0.005], *p* = 0.10) and indirect effect (estimate: -0.007, β = -0.006, *CI*_*0.95*_ = [-0.018–0.003], *p* = 0.16) were not significant.

The second simple mediation analysis, testing the mediating role of non-constructive ruminations, showed that both the direct effect (estimate: -0.234, β = − 0.03, *CI*_*0.95*_ = [-0.467–0.001], *p* = 0.049) and total effect were significant (estimate: -0.003, β = -0.03, *CI*_*0.95*_ [-0.059 – -0.003], *p* < 0.05) but not the indirect effect (estimate: -0.029, β = -0.03, *CI*_*0.95*_ = [-0.071–0.011], *p* = 0.153).

## Discussion

The primary objective of this study was to explore the combined role of insomnia, paranoia, anxiety, and non-constructive rumination. To this end, we conducted network analyses, complemented by subsequent mediation analyses.

The results revealed, for the first time to our knowledge, significant associations between daytime and nighttime symptoms of insomnia, anxiety, paranoid thoughts, and non-constructive rumination. Furthermore, they highlight significant differences in the interrelationships between paranoid thoughts and non-constructive ruminations depending on the presence or absence of insomnia.

Our first hypothesis proposed that network analyses would highlight stronger associations between anxiety, insomnia symptoms, non-constructive rumination, and paranoid thoughts in the insomnia group compared to the non-insomnia group. The results support this prediction. In the insomnia group, network analysis identified anxiety as the central node, strongly connected to paranoid thoughts, non-constructive rumination, and daytime insomnia symptoms. By contrast, in the non-insomnia group, the network displayed fewer and differently organized connections. Anxiety remained the most connected node, but it was directly linked to nighttime symptoms and both types of rumination, while paranoid thoughts were isolated from other variables. These results underline the crucial role of insomnia in fostering paranoid thoughts and non-constructive rumination, consistent with previous work^13^^14,15,21^. Indeed, depending on the presence or absence of insomnia among participants, the network structure varies considerably, isolating dysfunctional cognitions. Harrington & Cairney argued that sleep deprivation impairs working memory, favoring dysfunctional cognitions, which aligns with our findings^6^. Working memory deficits reduce an individual’s capacity to inhibit or shift away from maladaptive thought patterns, thereby increasing the likelihood of dysfunctional cognitions such as paranoid thoughts and non-constructive rumination.

Importantly, the network analysis showed that paranoid thoughts were more closely related to daytime symptoms than to nighttime symptoms. Daytime symptoms—such as fatigue, sleepiness, mood disturbances, and cognitive impairments—appear particularly influential. Harvey’s model of insomnia^8^ and Freeman’s model^15^ provide a useful explanation: sleep disturbances weaken cognitive control and increase negatively toned cognitive activity, which affects daily daytime functioning. This leads to daytime symptoms, manifested as elevated negative mood and altered attentional processes that promote biased interpretations of ambiguous situations.

In addition, anxiety emerged as an intermediary node between paranoid thoughts and nighttime symptoms, echoing prior findings^15,16^. Nocturnal symptoms—difficulty falling asleep, frequent awakenings, or early morning awakenings—are strongly associated with hypervigilance and anxious somatic arousal^31^. Such hyperarousal amplifies emotional reactivity, increasing the likelihood of threat interpretations and dysfunctional cognitions^8^. Thus, anxiety appears to act as a facilitator of paranoid ideation in individuals with insomnia.

Another important finding was the strong direct link between non-constructive rumination and paranoid thoughts, as well as an indirect link mediated by anxiety. This highlights the interconnection between emotional dysregulation and maladaptive cognition in insomnia^32^.

Our second hypothesis suggested that non-constructive rumination would mediate the insomnia–paranoia relationship in the insomnia group. The results confirmed this expectation in line with prior works^17,18^. To our knowledge, no earlier study had investigated the specific role of non-constructive rumination—a repetitive, abstract, and non-solution-oriented style of thinking^19^. We observed that this form of rumination was found to mediate the relationship only in the insomnia group, supporting evidence of its detrimental impact on mental health^20^. Here again, sleep disturbances may impair cognitive regulation, thereby fostering maladaptive thought patterns, which in turn promote paranoid thoughts^7^. However, given the cross-sectional nature of our data, this finding does not provide formal evidence of a causal relationship.

Our third hypothesis posited that anxiety would mediate the insomnia–paranoid thoughts link in both groups. This was only partially supported, as mediation emerged exclusively in the insomnia group. While anxiety is well known to correlate with both paranoid thoughts and insomnia^13,16,18^, its mediating effect appears to depend on the presence of sleep disturbances. Anxiety levels were not significantly different between groups, yet paranoid thoughts were more frequent among insomniacs, suggesting that insomnia amplifies the influence of anxiety on paranoid thinking.

Theoretically, these findings strengthen models linking psychopathology to sleep disturbances. Insomnia is indeed closely linked, particularly to suicide risk, and its association with psychotic symptomatology has also been documented^33,34^. They support the view of insomnia as a risk factor for dysfunctional cognitions and paranoid thoughts^7,15,16^.

Clinically, they highlight the potential of targeting insomnia through Cognitive-Behavioral Therapy for Insomnia (CBT-I) for the management of paranoid thoughts. Cognitive restructuring and thought control techniques specific to CBT-I help reduce non-constructive rumination by promoting more adaptive thinking patterns. In addition, the sleep improvements induced by CBT-I can decrease anxiety and emotional reactivity, thereby indirectly helping to alleviate paranoid thoughts. Previous studies have shown that CBT-I reduces psychopathological symptoms, including paranoid delusions in schizophrenia^35^, and recent meta-analyses confirm its efficacy in alleviating psychotic symptoms^36^.

However, some limitations must be acknowledged. Although the SCI is a validated tool for detecting insomnia in both general and clinical populations^22,25^, we did not conduct clinical interviews to confirm the diagnosis of insomnia. The sample was predominantly female, which is common in insomnia^37^ research and online surveys^38^ but may reduce generalizability, as psychopathological expressions can differ by gender^39^. Second, comorbid psychological or medical conditions were not assessed, which may have influenced the observed relationships. The use of an online survey also constitutes an important limitation due to the risk of sampling bias (self-selection bias, which may have led to a predominance of responses from women) and limits the representativeness of the population.

In conclusion, this study contributes to understanding the complex links between insomnia and paranoid thoughts. It demonstrates that daytime symptoms, rather than nocturnal symptoms, are most strongly associated with paranoia, and that anxiety and non-constructive rumination mediate this relationship among insomniacs. By identifying these mechanisms, the study emphasizes the importance of addressing sleep disturbances in clinical practice. Integrating CBT-I within treatment approaches may help reduce not only insomnia but also related cognitive and emotional dysfunctions commonly observed in psychiatric populations.

## Supplementary Information

Below is the link to the electronic supplementary material.

Supplementary Material 1

## Data Availability

The datasets used and/or analysed during the current study are available from the corresponding author on reasonable request.
