# Supplementary material for: Insomnia contributes to paranoid thoughts through mechanisms involving anxiety and non-constructive rumination
Source: Sci Rep. 2026 Feb 27;16:11324. doi: 10.1038/s41598-026-41689-8 (PMC13049045; doi:10.1038/s41598-026-41689-8)
Supplement: Supplementary file 1 — Supplementary Material 1 [file 41598_2026_41689_MOESM1_ESM.docx]

**SUPPLEMENTARY MATERIAL**

Table 1 : Correlations for the insomnia group (n=226) (above the diagonal) and the non-insomnia group (n=260) (below the diagonal).

| Variables | **1** | **2** | **3** | **4** | **5** | **6** | **7** |
| --- | --- | --- | --- | --- | --- | --- | --- |
| 1. Anxiety | - | -0.372** | -0.302** | -0.152** | -0.146* | 0.423** | 0.372** |
| 2. Insomnia (Total) | -0.322** | - | - | - | 0.127 | -0.250** | -0.284** |
| 3. Daytime symptoms | -0.160** | - | - | -0.089 | 0.078 | -0.251** | -0.273** |
| 4. Nighttime symptoms | -0.271** | - | -0.012 | - | 0.024 | -0.035 | -0.107 |
| 5. Constructive rumination | -0.276* | 0.175** | 0.193** | 0.071 | - | -0.079 | 0.071 |
| 6. Non-constructive rumination | 0.374** | -0.121 | -0.125* | -0.054 | -0.019 | - | 0.283** |
| 7. Paranoid thoughts | 0.128* | -0.136* | -0.050 | -0.150* | 0.071 | 0.143** | - |

** : p < 0.05* ; ** : *p < 0.01*
